# Maturational changes in frontal EEG alpha and theta activity from infancy into early childhood and the relation with self-regulation in boys and girls

**DOI:** 10.1016/j.dcn.2024.101445

**Published:** 2024-09-19

**Authors:** Marissa Hofstee, Joyce Endendijk, Jorg Huijding, Bauke van der Velde, Julie Vidal, Maja Deković

**Affiliations:** a Department of Clinical Child and Family Studies, Utrecht University, the Netherlands; b Department of Experimental Psychology, Helmholtz Institute, Utrecht University, Utrecht, the Netherlands; c Department of Developmental Psychology, Utrecht University, Utrecht, the Netherlands; d Laboratoire de Psychologie du Développement et de l′Éducation de l’enfant, UMR CNRS 8240, Université Paris Cité, Paris, France

**Keywords:** Brain maturation, Frontal cortex, Self-regulation, Delay of gratification, Early childhood, Sex differences

## Abstract

There is increasing interest in examining the development of frontal EEG power in relation to self-regulation in early childhood. However, the majority of previous studies solely focuses on the brain’s alpha rhythm and little is known about the differences between young boys and girls. The aim of the current study was therefore to gain more insight into the neural mechanisms involved in the emergence of self-regulation. The sample consisted of 442 children and data were collected at approximately 5 months, 10 months, and around 3 years of age. Latent growth curve models indicated that,while the neurobiological foundations of self-regulation are established during infancy,it is the maturation of the frontal alpha rhythm that contributes to variations in both observed and parent-reported self-regulation. In addition, it appears that boys might have a greater reliance on external regulation than girls during early childhood, as evident by higher scores of girls on both measures of self-regulation. More insight into the role of external regulators in brain maturation can help to implement interventions aimed at establishing bottom-up self-regulatory skills early in life, in order to provide the necessary foundations for the emergence of top-down self-regulatory skills in the preschool period.

## 1 Introduction

Early childhood is marked by a period of rapid maturational changes in brain structure and function. These changes include complex processes like neural pruning and myelination in the frontal cortex (Kolk and Rakic, 2022). The frontal cortex functions to facilitate self-regulatory skills, such as the ability to control emotions, behaviors, and cognition (Nigg, 2017, Zelazo et al., 2008). Previous studies demonstrate that the frontal cortex is among the latest brain regions to fully mature, with a maturation process that begins in early childhood and continues at a decreased rate into adolescence and early adulthood (Kolk and Rakic, 2022, Romine and Reynolds, 2005). This prolonged maturation process might explain the limited involvement of the frontal cortex in self-regulatory skills during the first years of life (for a meta-analysis on the relationship between frontal activity and self-regulation, see Hofstee et al., 2022a). However, higher-order mechanisms of self-regulation, such as executive functioning, are likely to emerge in children around the age of three, when the frontal cortex becomes sufficiently developed (Blair and Ursache, 2011, Garon et al., 2008). One theory of functional brain development is the maturational perspective, suggesting that the emergence of higher-order cognitive skills can be related to the maturation of underlying brain regions, including the frontal cortex (Johnson, 2001). Although research has focused on maturational changes in the brain in middle and late childhood (e.g., Cellier et al., 2021; Perone et al., 2018b), the critical period of early childhood remains relatively unexplored. Therefore, to provide a more comprehensive understanding of the neural mechanisms underlying the development of complex cognitive skills, the current study aimed to examine the relationship between maturational changes in the frontal cortex from infancy to early childhood and the development of self-regulatory skills in the preschool period.

### 1.1 Frontal EEG alpha and theta power

A valuable tool to assess maturational changes in the frontal cortex is baseline electroencephalography (EEG). Baseline EEG captures brain activity (i.e., power) within a specific frequency rhythm while children are awake and not engaged in any specific task (Anderson and Perone, 2018, Deco et al., 2013). In general, the alpha rhythm reflects inhibitory processes, such as filtering out task-irrelevant information (for a review, see Klimesch et al., 2007). Brain maturation in the alpha rhythm is assumed to consist of an increase in the amount of alpha frequency activity from infancy to early childhood (Bell and Fox, 1994; Cuevas and Bell, 2011; Marshall et al., 2002). While adults typically exhibit alpha activity in the frequency range of 9–12 Hz or a close derivative (e.g., 8–13 Hz), infants tend to show alpha activity in the lower frequency range of 6–9 Hz (Cuevas and Bell, 2022). However, given that the 6–9 Hz band could become limited in its utility for children at around 4 years of age, previous research underlines that the use of an extended frequency range, such as 7–10 Hz, may better capture alpha-type rhythms in the preschool period (Marshall et al., 2002). The theta rhythm is believed to serve as a mechanism through which neurons can facilitate and communicate top-down control across brain networks (Cavanagh and Frank, 2014). Young infants tend to show high levels of theta activity, but this activity is assumed to decrease with age from infancy into early childhood (Klimesch, 1999, Orekhova et al., 2006). The theta rhythm is characterized by activity in the frequency range of approximately 4–8 Hz in adults (Cavanagh and Frank, 2014) and 3–6 Hz in infants (Orekhova et al., 1999). Similar to the alpha rhythm, the peak frequency of the theta rhythm increases with age, reaching about 6 Hz in preschool children (Orekhova et al., 2006). As a result, previous research recommends the use of age-adjusted frequency bands to better capture maturational changes in both the alpha and theta rhythms (for a review, see Klimesch, 1999; Orekhova et al., 2006).

### 1.2 Relations between frontal EEG activity and self-regulation

Considering the critical period of maturational changes in the frontal cortex in the first years of life, it seems likely that the neurobiological basis of self-regulatory skills in preschool age children has its origins in infancy (Whedon et al., 2020). For example, longitudinal research in early childhood showed that variations of baseline frontal alpha power at 10 months of age were associated with individual differences in performance on a composite of self-regulation tasks in children at age 4 (Kraybill and Bell, 2013). In addition, baseline frontal alpha power at 5 months of age has been found to be positively related to self-regulation observed from a composite of self-regulation tasks at 4 years of age (Broomell et al., 2020). This indicates that maturational changes in frontal EEG activity during the first years of life may contribute to the emergence of higher order mechanisms of self-regulation in the preschool period. Yet, the majority of developmental research focuses on maturational changes in EEG activity in children above 3 years of age (e.g., Perone et al., 2018a, Perone et al., 2018b; Cellier et al., 2021), a time when higher-order self-regulatory skills typically develop (Garon et al., 2008, Hendry et al., 2016). One of the few studies that examined the developmental trajectories of baseline frontal EEG activity during the first year of life in relation to child self-regulation showed that maturational changes in frontal alpha activity were not related to variations in self-regulation in infants observed from the A-not-B task (MacNeill et al., 2018). However, different results were found in a study focusing on the preschool period. That is, while initial levels of baseline frontal alpha power at 10 months of age were not associated with self-regulation (observed from a battery of inhibitory control tasks) at 4 years of age, greater increases in frontal alpha power from 10 months to 4 years of age were associated with higher levels of children’s self-regulation at age 4 (Whedon et al., 2020). This implies that, although the neurobiological basis for self-regulation is laid in infancy, it is the maturation of the frontal cortex during early childhood that predicts the variations in self-regulatory skills across the preschool period. Nevertheless, there is limited evidence for this notion and it is unclear if maturation in the alpha rhythm is also related to self-regulation measured by parent-reported questionnaires, that might reflect another form of self-regulation. Recently, a meta-analysis revealed that scores on executive functioning tasks and parent-reported measures of self-regulation were inversely related to frontal alpha power (Hofstee et al., 2022a). That is, lower levels of frontal alpha power were related to higher levels of parent-reported self-regulation, whereas higher levels of frontal alpha power were related to higher levels of self-regulation as assessed by executive functioning tasks. Executive functioning tasks, such as the delay of gratification task, generally assess top-down forms of self-regulation, including higher-order cognitive functions that engage, direct, or coordinate reactive (bottom-up) processes (Perone et al., 2018a, Zelazo et al., 2008). Specifically, the delay of gratification task was designed to examine children’s ability to suppress impulsive behaviors and resist the temptation of immediate reward (Mischel et al., 1989).

In contrast, parent-reported questionnaires on self-regulation in young children tend to include bottom-up regulatory processes that are often influenced by external regulating factors, such as parenting behaviors (Bernier et al., 2010, Gartstein and Rothbart, 2003). For instance, the Early Childhood Behavior Questionnaire provides a different perspective on self-regulation than the delay of gratification task by assessing temperament traits of self-regulation, such as attention focusing (i.e., the capacity to maintain attention on a task or object) and perceptual sensitivity (i.e., the ability to detect subtle stimuli from the environment; (Putnam et al., 2006). These different measures of self-regulation may be uniquely associated with variations in frontal EEG activity (Hofstee et al., 2022a). Thus, gaining more insight into the neural mechanisms involved in both top-down and bottom-up self-regulation processes is crucial for a more profound understanding of how self-regulatory skills emerge in the preschool period.

In contrast to more broader developmental EEG research examining the theta rhythm, and other frequency rhythms such as gamma (e.g., Tierney et al., 2012; Tomalski et al., 2013), the theta rhythm and its relationship with self-regulatory processes has received little attention. Moreover, the few studies on frontal theta activity mainly examined children's EEG recordings during the performance of a self-regulation task (e.g., Orekhova et al., 1999; Stroganova et al., 1998). However, EEG assessments of brain activity are heavily influenced by cognitive demands, leading to contrasting patterns of EEG activity when comparing test conditions with baseline conditions (for a review, see Klimesch, 1999). In the context of baseline EEG, findings of the study of Perone and Gartstein (2019) revealed that lower levels of frontal theta power were related to higher levels of parent-reported self-regulation in infancy. More research is needed to determine whether maturational changes in theta power values also predict self-regulation at later ages, as well as self-regulation observed from an executive functioning task. This can provide a more comprehensive picture of the neural mechanisms underlying self-regulation than measures of frontal alpha power alone (Whedon et al., 2020).

### 1.3 Differences between boys and girls

Earlier studies also suggest that children’s sex can be indicative of differences in brain maturational processes (Gartstein et al., 2020). For example, greater maturational changes in the frontal cortex have been found in girls compared to boys. Specifically, at 4 years of age, girls showed higher levels of frontal alpha power during a self-regulation task in comparison to boys (Cuevas et al., 2016). This might indicate that girls manifest a more mature pattern of functional brain development during early childhood than boys. Importantly, young girls also typically show higher levels of self-regulation than boys, such as better abilities to control inappropriate responses and behaviors (for a meta-analysis, see Else-Quest et al., 2006). Girls also tend to outperform boys with regard to self-regulation observed from delay of gratification tasks (for a meta-analysis, see Silverman, 2021). These higher levels of self-regulatory skills in girls might be related to greater brain maturation in girls than boys in early childhood (Cuevas et al., 2016; Gartstein et al., 2020).

Nevertheless, there remains a lack of research on sex differences concerning brain maturation from infancy to early childhood. Gaining more insight into sex differences in the relation between brain maturation and child self-regulation is crucial for a better understanding of frontal alpha and theta activity as indicators of brain maturation during early childhood (Langrova et al., 2012). Therefore, the current study fills in this gap by examining sex differences in the context of brain maturation and child self-regulation, contributing to a deeper understanding of the development of self-regulation and offering potential insights for the enhancement of interventions aimed at fostering positive developmental trajectories in both boys and girls.

### 1.4 The current study

An important goal in developmental neuroscience is to disentangle the complex link between the emergence of cognitive functions and the underlying maturational changes in brain structure and function (Johnson, 2001). In recent years, great progress has been made in examining the developmental trajectories of frontal power underlying the emergence of self-regulation in infancy and early childhood (e.g., MacNeill et al., 2018; Whedon et al., 2020). However, as the majority of previous studies solely focuses on the alpha rhythm, to our knowledge, it is currently unknown how maturation of frontal theta power from infancy to early childhood is related to the emergence of self-regulation in the preschool period. In addition, little is known about the maturational differences in baseline EEG activity underlying self-regulatory skills between young boys and girls.

Gaining more insight into the neural mechanisms involved in the development of self-regulatory skills can facilitate interventions that specifically target the neural processes associated with cognitive development. Therefore, the current study aimed to examine (1) the maturational changes in frontal EEG alpha and theta activity from infancy into early childhood, taking into account the evolution of the frequency bands with age, (2) the relationship between the initial levels and the maturational changes in frontal EEG alpha and theta activity with self-regulation, both observed from a self-regulation task (i.e., the ability to delay gratification) and reported by parents in a questionnaire (i.e., effortful control), and (3) sex differences in frontal EEG alpha and theta activity and child self-regulation, as well as in the association between maturational changes in frontal EEG alpha and theta activity and child self-regulation.

First, based on previous studies (e.g., Cuevas and Bell, 2022; Klimesch, 1999; Marshall et al., 2002), it was expected that frontal EEG alpha power values would increase, whereas frontal EEG theta power values would decrease from infancy to early childhood. Second, it was expected that higher initial alpha power and lower initial theta power levels, as well as an increase in frontal alpha power and a decrease in frontal theta power would be related to higher levels of self-regulation (both the ability to delay gratification and effortful control). Third, it was expected that girls would show higher initial levels of alpha power, lower initial levels of theta power values, a stronger increase in alpha power, and a stronger decrease in theta power (i.e., greater brain maturation) compared to boys, as well as higher levels of self-regulation.

## 2 Method

### 2.1 Participants

Data for the current study were derived from three waves of the YOUth Baby & Child cohort (T1 = 5 months, T2 = 10 months, T3 = 3 years). The YOUth Baby & Child cohort is a large-scale ongoing longitudinal study following children from 20 weeks gestational age until the age of 6 years, conducted in the Netherlands. More information about the design and procedure of the YOUth study can be found in Onland-Moret et al. (2020). Children were included if they had participated at the third wave at the start of the current study, which resulted in a sample of 470 children. Within this sample, 28 children had missing EEG data on all three waves as a result of attrition due to fussiness, cap refusal, or data loss (for more information about EEG data attrition within the YOUth cohort study, see Van der Velde and Junge, 2020). These children were excluded from the current study. None of the children had missing data on both self-regulation outcomes. Therefore, the final sample consisted of 442 children (52.5 % girls).

Mean age of the children was 23.72 weeks (*SD* = 3.60) at T1, 45.68 weeks (*SD* = 3.96) at T2, and 177.81 weeks (*SD* = 43.86) at T3. The flexible longitudinal design of the current study (e.g., broad age ranges) is assumed to provide more detailed information on developmental processes over time (Lane and Kelleher, 2023). All children were born full-term (38–42 weeks), had normal birth weight, and no developmental delays or abnormalities in visual or auditory processing. In addition, the majority of the children in the current sample came from relatively high SES families, as indicated by 56 % of the mothers having at least a bachelor’s or master’s degree and 68 % of the families reporting a monthly gross household income that was above €4000). Moreover, 89 % of the children were Dutch, while less than 2 % were from non-European countries.

### 2.2 Procedure

At each measurement wave, participants visited the research laboratory of YOUth at Utrecht University, the Netherlands (Onland-Moret et al., 2020). During the lab visits, both behavioral and cognitive development of the children were measured through various tasks (e.g. EEG, self-regulation tasks). The data were collected under the guidance of trained and experienced researchers and research assistants. In addition, parents received several online questionnaires at each measurement wave. The parent-reported questionnaires about the behaviors of the child were filled out by the primary caregiver of the child. The study was approved by The Medical Research Ethics Committee of the University Medical Center Utrecht (METC 14-616) and both parents provided written informed consent at each measurement wave. Parents were compensated €30 for each lab visit. A more detailed description of the collected data during each wave of the YOUth study is available at: https://www.uu.nl/en/research/youth-cohort-study.

### 2.3 Measures

#### 2.3.1 Frontal EEG power

The EEG recording consisted of a baseline setting (i.e., quiet wakefulness) that was acquired in a quiet and dimly lit room at all three waves. Children were seated on their parents’ lap or in a car seat positioned at eye level 65 cm from the computer screen. During the baseline physiology, children passively watched 60-second videos of singing women and moving toys without human interference, which is a common procedure for a baseline period in young children (Cuevas and Bell, 2022). These videos were repeated three times, with short breaks in between. EEG was recorded using a cap with 32 electrodes (ActiveTwo system, BioSemi) positioned according to the international 10/20 system. Recordings were made from the frontal pole (Fp1 and Fp2), medial frontal (F3 and F4), lateral frontal (F7 and F8), central (C3 and C4), temporal (T7 and T8), medial parietal (P3 and P4), lateral parietal (P7 and P8), and occipital (O1 and O2) sites and sampled at a rate of 2048 Hz. The Common Mode Sense (CMS) and Driven Right Leg (DRL) electrode were used to provide an active ground. The EEG recordings were analyzed in Matlab, using functions of the FieldTrip toolbox (Oostenveld et al., 2011). More specific information on the EEG analysis, including the use of age-adjusted frequency ranges, can be found in the Supplementary materials.

#### 2.3.2 The ability to delay gratification

The gift-delay task (Kochanska et al., 1996) was used to assess children's capacity to delay gratification in response to an attractive stimulus at T3. Children were seated at a table and the experimenter presented each child with a bag that contained a wrapped gift. Subsequently, the children were told to wait in their chair and not to touch or open the bag with the gift until the examiner returned with a bow. Then, the experimenter left the room and returned after 180 s. Parents were instructed to stay in the room and to remain as neutral as possible. All sessions were videotaped and later coded by two trained coders. Interrater reliability between the two coders was excellent, with ICCs ranging from .91 to .99 More specific information on the coding of the videos can be found in the Supplementary materials.

#### 2.3.3 Effortful-control

Parent-reported self-regulation (i.e., effortful control) at T3 was assessed using the Early Childhood Behavior Questionnaire – short form (ECBQ-SF; (Putnam et al., 2006) and Children’s Behavior Questionnaire – very short form (CBQ-SF; (Putnam and Rothbart, 2006), depending on the age of the child (ECBQ-SF for children < 3 years at T3 and CBQ-SF for children 3 years of age and older at T3). The self-regulatory behaviors of the children were rated on a 7-point scale ranging from *never/extremely untrue* (1) to *always/extremely true* (7). An example item is “Can easily stop doing something when told no”. For the ECBQ-SF, effortful control was determined by averaging the scores on the Attention Focusing (*N* = 6 items), Attention Shifting (*N* = 8 items), Cuddliness (*N* = 6 items), Inhibitory Control (*N* = 6 items), and Low-Intensity Pleasure (*N* = 6 items) subscales (Putnam et al., 2006). For the CBQ-SF, the subscales Attention Focusing (*N* = 6 items), Inhibitory Control (*N* = 6 items), Perceptual Sensitivity (*N* = 6 items), and Low Intensity Pleasure (*N* = 8 items) were averaged (Putnam and Rothbart, 2006). The items used to create the self-regulation scores had good internal consistency (α = .86 for the ECBQ-SF and α = .81 for the CBQ-SF).

### 2.4 Data-analysis

Latent growth curve modeling was conducted in Mplus 8.8 to estimate individual differences in the maturational changes in frontal alpha and theta power separately (Muthén and Muthén, 2017). First, unconditional growth models were modelled. Due to the fact that there were three measurement waves in the current study, the models were constrained to solely estimate linear slopes. Because children varied substantially in age at each measurement wave, the TSCORES option in Mplus was used to allow time to act as a covariate on the growth models (Muthén and Muthén, 2010). The TSCORES option takes into account the age differences at each measurement wave by including age as a defining variable to scale the factor loadings and estimate the growth curve, rather than defining growth with fixed factor loadings. The inclusion of random effects accounts for the extent to which the estimates deviate from the fixed-effect estimate for each individual, thereby enabling unique growth trajectories for all children (McNeish and Matta, 2018). In this way, all children contribute to the estimation of parts of the growth trajectory for the ages at which they provided data (Mehta and West, 2000).

**Fig. 1:**
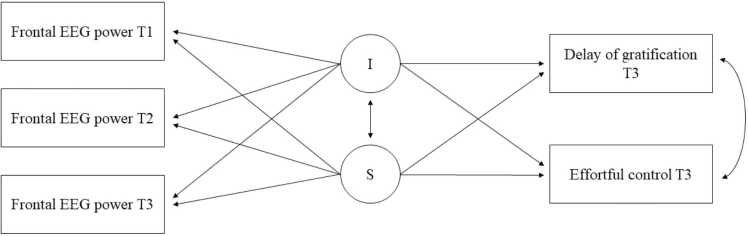
*The hypothesized model to test the relation between the maturational changes in frontal EEG power and both the ability to delay gratification and effortful control. Note.* I = intercept (initial level), S = slope (rate of growth). The model was run for the alpha and theta rhythm separately.

Next, in the two separate conditional growth models of frontal alpha and theta power, both the ability to delay gratification and effortful control were added simultaneously as outcome variables (Fig. 1). The ability to delay gratification and effortful control were allowed to correlate with each other within the models. In addition, given the broad age ranges in the YOUth cohort, age at T3 was included as a covariate on the outcome variables within the conditional growth models. It was intended to use bootstrap methods to acquire more robust model estimates. However, Mplus does not allow the use of bootstrap methods in combination with random slopes and multi-group analyses. Therefore, it was necessary to deviate from the pre-registered analysis plan and to run the models without the use of bootstrap methods.

Subsequently, sex differences in the developmental growth trajectories and their association with individual differences in self-regulation were tested using multigroup analyses with children’s sex as the grouping variable. A constrained growth model in which all parameters were constrained to be equal across groups was compared to an unconstrained growth model in which all parameters were freely estimated. If the unconstrained model was a better fit to the data compared to the constrained model, this suggested the presence of differences between boys and girls (Muthen and Muthen, 2012). When using the TSCORES option in Mplus, the standard fit indices are not available. As a result, only the Akaike Information Criterion (AIC) and Bayesian Information Criterion (BIC) were used to compare the model fit of the constrained and the unconstrained growth models. Lower AIC and BIC values suggest a better fit to the data, with decreases in values larger than 10 indicating a serious improvement in model fit (Raftery, 1995, Vrieze, 2012).

#### 2.4.1 Missing data

Most children (71.7 %) had at least two time points of EEG data, but only 27.8 % of the children had EEG data on all three time points. In addition, 3.8 % of the children had missing data on the gift delay task and 19.9 % of the parents did not fill in the questionnaire on child self-regulation. Little’s MCAR test (Little, 1988) demonstrated that the missing data was not missing at random (MNAR), χ^2^ = 82.732, *p* = .004. Therefore, the Full-information maximum likelihood method could not be applied to deal with the missing data in the current study.

Multiple imputation (MI) is an alternative method that creates several plausible complete versions of the incomplete data set and combines the results of the statistical analyses of the complete datasets into an overall statistical analysis (Rubin, 1987, Van Buuren, 2018). Given that MI produces less biased estimates than listwise deletion when data are MNAR (Van Ginkel et al., 2020), MI was applied to deal with the missing data in the current study. By using all available information (e.g., outcome variables and demographics), 15 imputed datasets were generated using Bayesian analysis and all were analyzed (Rubin, 1987). Subsequently, the parameter estimates were averaged over the imputed datasets and the standard errors were calculated using the average over the imputed datasets and between analysis parameter estimate variation (Muthén and Muthén, 2017). The hypotheses, study design, and planned analyses were preregistered at the Open Science Framework (https://osf.io/hvzqw).

## 3 Results

### 3.1 Descriptive statistics

Table 1 presents the means and standard deviations for boys and girls separately. The descriptive statistics of frontal EEG activity without age-specific frequency ranges can be found in Table S1. One outlier was identified on effortful control and was believed to be unrepresentatively low. Therefore, this outlier was winsorized by giving it a marginally lower value than the most extreme not outlying value (lowest non-outlying number + difference between lowest non-outlying number and before lowest non-outlying number; Tabachnick and Fidell, 2013). Pearson correlation analyses were performed with the winsorized and nonwinsorized data and the winsorization of the outlier did not lead to different results. Therefore the results of the winsorized data are presented. In line with the assumption that skewness and kurtosis values between − 2 and 2 are considered acceptable for the assumption of normality (George and Mallery, 2010), all variables were normally distributed.

**Table 1:** Descriptive statistics for boys and girls.

	Boys (*N* = 210)		Girls (*N* = 232)	
	*M*	*SD*	*M*	*SD*
1. Frontal alpha power T1	0.88	0.16	0.91	0.17
2. Frontal alpha power T2	0.76	0.17	0.77	0.14
3. Frontal alpha power T3	0.83	0.16	0.84	0.19
4. Frontal theta power T1	0.75	0.12	0.78	0.12
5. Frontal theta power T2	0.85	0.11	0.86	0.10
6. Frontal theta power T3	0.78	0.30	0.81	0.29
7. Delay of gratification behavior	3.60	1.29	3.91	1.16
8. Observed latency score	88.53	66.54	101.33	67.29
8. Effortful control	5.11	0.61	5.27	0.63

*Note*. To enhance the interpretability of the descriptive statistics, the mean scores for both the behaviors during the delay of gratification task and the latency to exhibit this behavior are reported, rather than the composite score. In addition, the descriptive statistics of the non-transformed power values are reported.

On average, there was a difference between boys and girls regarding their self-regulation scores at T3. Girls scored significantly higher on the ability to delay gratification*, t*(432) = 2.71, *p* = .007, as well as on effortful control, *t*(352) = 2.41, *p* = .016, compared to boys. The Cohen's d was 0.26 for both measures of self-regulation, indicating a small difference in self-regulation between boys and girls. However, boys and girls did not significantly differ in their frontal alpha activity at T1, *t*(264) = 1.65, *p* = .101, T2, *t*(284) = 0.99, *p* = .322, and T3, *t*(328) = 0.44, *p* = .658. Regarding differences in frontal theta activity, girls showed significantly higher frontal theta power values at T1 than boys*, t*(264) = 2.09, *p* = .038. Yet, this consisted of a very small difference between boys and girls (*d* = 0.16). No significant differences in frontal theta power values were observed between boys and girls at T2, *t*(284) = 1.01, *p* = .315, and T3, *t*(328) = 1.08, *p* = .283.

### 3.2 Direct relations between the study variables

Results of the Pearson correlations are presented in Table 2. The results showed that greater abilities to delay gratification were related to higher levels of effortful control. However, the correlation between these two variables was relatively low (*r* = .14). Frontal alpha power was positively related to self-regulation in the preschool period, but not during infancy. More specifically, higher levels of frontal alpha power at T3 were associated with higher levels of both the ability to delay gratification and effortful control at T3.

**Table 2:** Correlations for study variables.

	1	2	3	4	5	6	7	8
1. Frontal alpha power T1	—							
2. Frontal alpha power T2	.20^**^	—						
3. Frontal alpha power T3	.13	.27^**^	—					
4. Frontal theta power T1	.63^**^	.24^**^	.02	—				
5. Frontal theta power T2	.19*	.55^**^	.06	.35^**^	—			
6. Frontal theta power T3	.12	− .14*	− .06	.08	− .02	—		
7. Delay of gratification (composite)	− .09	− .06	.17^**^	− .03	− .06	− .13*	—	
8. Effortful control	− .09	− .03	.15*	− .15*	− .08	.00	.14^**^	—

*Note.* *p < .05. ^**^p < .01.

In contrast, frontal theta power at T1 was negatively related to effortful control. In addition, frontal theta power at T3 was negatively related to the ability to delay gratification at T3. However, no relations were found between frontal theta power at T2 and T3 and effortful control at T3. The results with regard to frontal EEG activity without age-specific frequency ranges can be found in Table S2.

### 3.3 Maturational changes in frontal EEG activity

To assess the changes in frontal EEG activity over time, unconditional latent growth curves for frontal alpha power and frontal theta power were modelled. The results showed that, on average, frontal alpha power remained stable across infancy and early childhood (slope = − 0.001, *p* = .540). Children significantly differed in their initial levels of frontal alpha power (intercept = 0.005, *p* = .006). Importantly, the variance of the random slopes of frontal alpha power approached zero. Therefore, the residual variances of the random slopes of frontal alpha power were restricted to zero, which is a common procedure to make model estimation possible (Muthen and Muthen, 2012). When fixing the residual variance of the slope to zero, the slopes themselves can still vary as a function of the inclusion of covariates. This might be due to a higher power to detect slope variability when covariates are included (Hertzog et al., 2006, LaHuis and Ferguson, 2009, Stoel et al., 2006). Therefore, the random slopes of frontal alpha power were still included in further analyses. Associations between the intercept and the slope of frontal alpha power were not estimated as the variance of the random slope was fixed to zero.

Similar to frontal alpha power, frontal theta power generally remained stable over time from infancy into early childhood (slope = − 0.003, *p* = .116). Children showed significant differences in their initial levels of frontal theta power (intercept = 0.007, *p* = .009), as well as in their rate in change over time (slope = 0.001, *p* = < .001). The intercept and slope of frontal theta power were negatively correlated (*r* = - 0.002, *p* = < .001), indicating that for children with a higher starting point of frontal theta power, this was associated with a lower rate of change.

### 3.4 Relations between frontal EEG activity and self-regulation

Subsequently, the relationship between the intercept and linear slopes of frontal EEG alpha and theta power with both the ability to delay gratification and effortful control were examined in two separate conditional models. The corresponding results^1^ are reported in Table 3. Results indicated that, although the initial levels of frontal alpha power were not associated with variations in child self-regulation, maturational changes in frontal alpha power from infancy into early childhood were positively related to both the ability to delay gratification and effortful control in preschool age children. More specifically, children who showed greater increases in baseline frontal alpha power had higher levels of self-regulation in the preschool period. With regard to the theta rhythm, the intercept and the slope of baseline frontal theta power were unrelated to both the ability to delay gratification and effortful control.

**Table 3:** Unstandardized estimates of the LGCM.

Model parameters	*B*	*SE*	*p*	95 % CI
Intercept frontal alpha power → delay of gratification	− 0.34	0.25	.175	[− 0.83, 0.15]
Slope frontal alpha power → delay of gratification	**2.66**	**0.96**	**.006**	**[0.78, 4.53]**
Intercept frontal alpha power → effortful control	− 0.36	0.27	.193	[− 0.90, 0.18]
Slope frontal alpha power → effortful control	**2.69**	**1.07**	**.012**	**[0.59. 4.79]**
Intercept frontal theta power → delay of gratification	− 0.22	0.31	.479	[− 0.83, 0.39]
Slope frontal theta power → delay of gratification	− 0.01	0.65	.987	[− 1.29. 1.27]
Intercept frontal theta power → effortful control	− 0.82	0.74	.265	[− 2.27, 0.63]
Slope frontal theta power → effortful control	− 1.06	1.50	.481	[− 4.00. 1.88]

*Note.* Estimates presented in bold refer to statistically significant estimates.

### 3.5 Differences between boys and girls

To further examine possible sex differences in the development of frontal alpha and theta activity between boys and girls, multigroup analyses were performed. The results indicated that the unconstrained model and the constrained model of the development of both frontal alpha and theta power yielded identical model fit indices (see Table 4). These findings suggest that the development of frontal alpha and theta power follow a similar pattern in terms of the initial level and growth rate for boys and girls throughout infancy and early childhood.

**Table 4:** Fit indices of the multi-group analysis.

	Constrained		Unconstrained	
	AIC	BIC	AIC	BIC
Unconditional model of frontal alpha power	− 512.434	− 463.338	− 512.434	− 463.338
Unconditional model of frontal theta power	− 282.756	− 217.295	− 282.756	− 217.295
Conditional model of frontal alpha power	− 2268.408	− 2174.308	− 2288.697	− 2149.593
Conditional model of frontal theta power	− 2038.679	− 1932.305	− 2056.093	− 1900.623

Next, sex differences in the relation between the development of frontal EEG activity and self-regulation were examined. The comparison between the unconstrained and constrained models of frontal alpha activity revealed that the unconstrained model had a lower AIC value, indicating the presence of sex differences between boys and girls.^2^ The model results showed that the initial levels of frontal alpha power were unrelated to the ability to delay gratification and effortful control in both boys and girls (see Table 5). However, sex differences emerged concerning the link between changes in frontal alpha power over time and variations in self-regulation. More specifically, increases in frontal alpha power were associated with greater abilities to delay gratification in girls, but not in boys, whereas increases in frontal alpha power were associated with higher levels of effortful control in boys, but not in girls.

**Table 5:** Unstandardized estimates of the unconstrained LGCM of the multi-group analysis.

	Boys (*N* = 210)			Girls (*N* = 232)		
	*B*	*SE*	*p*	*B*	*SE*	*p*
Intercept frontal alpha power → delay of gratification	− 0.35	0.34	.338	− 0.60	0.98	.542
Slope frontal alpha power → delay of gratification	2.25	1.28	.078	**3.19**	**1.36**	**.019**
Intercept frontal alpha power → effortful control	− 0.56	0.37	.125	− 0.17	0.75	.822
Slope frontal alpha power → effortful control	**3.85**	**1.54**	**.013**	2.06	1.46	.160
						
Intercept frontal theta power → delay of gratification	− 0.26	0.81	.746	− 0.26	0.34	.439
Slope frontal theta power → delay of gratification	− 0.31	1.81	.863	0.16	0.67	.815
Intercept frontal theta power → effortful control	− 1.39	2.64	.599	− 0.56	0.62	.367
Slope frontal theta power → effortful control	− 2.36	6.09	.699	− 0.57	1.15	.624

*Note.* Estimates presented in bold refer to statistically significant estimates.

For frontal theta power, the unconstrained model showed lower AIC and BIC values compared to the constrained model, indicating better model fit. However, the model results revealed that boys and girls did not differ in the relation between the development of frontal theta power and self-regulation during infancy and early childhood. That is, maturational changes in frontal theta power were unrelated to the ability to delay gratification and effortful control in both boys and girls.

## 4 Discussion

The current study examined the maturational changes in baseline frontal EEG alpha and theta power from infancy into early childhood and the associations with individual differences in self-regulation across the preschool period in both boys and girls. The findings demonstrated that increases in frontal alpha power from infancy into early childhood were positively related to variations in self-regulation in the preschool period. Importantly, increases in frontal alpha power were associated with greater abilities to delay gratification in girls, but with higher levels of effortful control in boys. Despite direct negative relations between theta power and child self-regulation, the maturational changes in frontal theta power were unrelated to both measures of self-regulation across the preschool period.

### 4.1 Development of frontal EEG alpha and theta power

Based on previous research findings (e.g., Klimesch, 1999; Marshall et al., 2002), it was expected that alpha rhythm activity would increase over time, whereas theta rhythm activity would decrease over time. Surprisingly, the current study found that frontal alpha and theta power in baseline EEG generally remained stable from infancy to preschool years. One explanation for these discrepant findings may be that studies on maturational changes in baseline frontal EEG power solely focused on infancy (MacNeill et al., 2018) or predominantly used non-age-adjusted frequency ranges (Whedon et al., 2020). The approach of using non-age-adjusted frequency ranges potentially overestimates alpha activity and underestimates theta activity in older children, given the increase in theta rhythm peak frequency towards about 6 Hz in preschoolers (Orekhova et al., 2006). The stability observed in frontal EEG activity in the current study may therefore be attributed to the use of age-adjusted frequency bands, as the upward shift in frequency ranges accounts for age-related increases in peak frequencies (Klimesch, 1999, Orekhova et al., 2006). This stability might be specific to early childhood, as older children with individually adjusted frequency bands demonstrated varying patterns of increases and decreases in frontal alpha and theta power in previous research (Perone et al., 2018b).

### 4.2 Relations between frontal EEG activity and self-regulation

In line with the findings of a meta-analysis (Hofstee et al., 2022a), the results of the current study showed that frontal alpha power was related to self-regulation in the preschool age period, while no relations were found with frontal alpha power during infancy. This suggests that the frontal cortex is still in the early stages of maturation during the first year of life and may not yet be important for self-regulatory behaviors in the preschool period (Blair and Raver, 2015). However, it is important to note that previous studies using other self-regulation tasks, such as the A-not-B task, have demonstrated a relation between frontal alpha power and self-regulation in infancy (Bell, 2001). This indicates that crucial brain maturation processes are already occurring during this developmental stage. This was also reflected by our findings that maturational changes in frontal alpha power during the first years of life were positively related to both the ability to delay gratification and effortful control in preschool age children. The findings of the current study therefore align with the maturational perspective of Johnson (2001), suggesting that the emergence of higher-order cognitive skills can be related to the maturation of underlying brain regions, including the frontal cortex. Regarding the theta rhythm, it was expected that lower levels of frontal theta power would be related to higher levels of child self-regulation. Indeed, lower levels of theta power at 5 months of age were related to higher levels of effortful control around 3 years of age. In addition, lower levels of theta power around 3 years of age were related to greater abilities to delay gratification around 3 years of age. In the first years of life, children gradually shift from more bottom-up forms of self-regulation to more top-down forms of self-regulation (Blair and Ursache, 2011, Nigg, 2017). These results therefore align with the idea that parent-reports of self-regulation include numerous items that capture bottom-up self-regulation mechanisms, such as external regulation, that begin to emerge early in life (e.g., around 5 months of age). In contrast, more top-down forms of self-regulation (i.e., delay of gratification) might rely on activity in the frontal cortex emerging later in early childhood (Blair and Ursache, 2011, Nigg, 2017).

Despite the small but significant direct relations between frontal theta power and self-regulation, the current study showed that the maturational changes in theta power from infancy into the preschool period were unrelated to both measures of self-regulation. Recently, Cuevas and Bell (2022) proposed that young children typically show a decrease in the amount of low frequency activity (i.e., lower-theta rhythm), but an increase in the amount of intermediate frequency activity (i.e., upper-theta rhythm) over time. Subsequently, the upper-theta rhythm will increasingly demonstrate properties similar to the alpha rhythm (Orekhova et al., 2006). A possible explanation for the null finding might therefore be that the traditional EEG band analysis (e.g., infant and adult bands) leads to overly wide boundaries, in which the variations in age-related changes might be blurred (Klimesch, 1999). Yet, to date, the field of developmental EEG research lacks standardization regarding the use of narrower frequency boundaries, emphasizing the need for further exploration in this direction.

### 4.3 Differences between boys and girls

In the current study, the ability to delay gratification and effortful control were not highly related, indicating that both measures may focus on different aspects of self-regulation (Hofstee et al., 2022b, Nigg, 2017). However, in line with meta-analyses on sex differences in both the ability to delay gratification and effortful control (Else-Quest et al., 2006, Silverman, 2021), the current study revealed that girls outperformed boys significantly on both self-regulation measures. These results suggest a certain degree of consistency in sex differences across various self-regulation assessments. Higher scores on both the ability to delay gratification and effortful control indicate that girls demonstrate superior abilities at the same age in both top-down and bottom-up self-regulation processes. Furthermore, it suggests that boys may rely more on external regulatory factors during their early development than girls (Blair and Ursache, 2011, Nigg, 2017). Insight into the mechanisms underlying these differences between boys and girls can help caregivers to tailor their support and guidance to meet the distinct needs of both girls and boys as they develop crucial self-regulation skills. In contrast to prior research showing small but significant differences in frontal alpha power between boys and girls (Cuevas et al., 2016), boys and girls did not differ in their levels of frontal alpha activity across all three waves in the current study. In addition, no differences emerged in frontal theta power values around 10 months and 3 years of age. However, surprisingly, girls showed higher levels of frontal theta power than boys around 5 months of age, which is generally considered as a sign of immaturity (Orekhova et al., 2006). The findings of the current study therefore suggest that girls initially show less matured patterns of brain activity compared to boys. Yet, given that there were no sex differences in the maturational changes in frontal theta power, these sex-related differences seem to disappear with increasing age and even reverse to girls exceeding boys in self-regulatory abilities at a later age.

The maturational changes in frontal alpha power were related to the ability to delay gratification in girls, but not in boys. This might suggest that the frontal cortex of girls matures earlier than the frontal cortex of boys, allowing them to engage in top-down self-regulatory processes like delay of gratification skills during the preschool period (Blair and Ursache, 2011). However, the significant sex differences that were found in the current study were minimal, given that the development of frontal alpha power showed a trend with the ability to delay gratification in boys (*p* = .078). In addition, the differences in maturity between boys and girls were not reflected in the changes of frontal alpha and theta power over time. Previous research underlines the strong relationship between EEG power and other brain measures, including measures of functional connectivity (Demuru et al., 2020). Therefore, future research incorporating additional measures of brain maturation is needed to further elucidate possible sex differences in early childhood.

In contrast to the ability to delay gratification, the developmental increases in frontal alpha power were linked to higher levels of effortful control in boys, but not in girls. To date, the factors contributing to these sex differences remain unclear and a comprehensive understanding would require a more extensive and in-depth examination. A possible explanation for the small differences might be that, although there are multiple items of the parent-reported questionnaire (ECBQ) that capture top-down forms of self-regulation (Gartstein and Rothbart, 2003, Hofstee et al., 2022b), the (E)CBQ still includes numerous items that capture forms of external self-regulation (e.g., “Likes to cuddle up to his/her caregiver”). Consequently, effortful control as assessed by a parent-reported questionnaire might be considered as a more reactive (bottom-up) mechanism of self-regulation compared to top-down self-regulation measures like the delay of gratification task (Hofstee et al., 2022a). These findings suggest that future studies could gain insights by focusing on more specific subscales, such as inhibitory control, which are more closely associated with top-down self-regulation (Cuevas et al., 2012).

Previous research demonstrated that the frontal cortex is involved in perceiving invariance or novelty of the immediate environment and that there might be a reduction in activation when learned tasks or behaviors become automatic (Saling and Phillips, 2007). For example, frontal activity tends to decrease when children become familiar with a stimulus through repetition in early childhood (Nakano et al., 2009). Considering that girls showed higher levels of self-regulation compared to boys, but the maturational changes in the frontal cortex were solely associated with effortful control in boys, it may be that the self-regulatory behaviors in the home environment have become more routine and automatic within girls. However, we have found no clear evidence for the underlying neural mechanisms accounting for these sex differences in child self-regulation. Nevertheless, the findings emphasize the importance of caregivers to act as external regulators for boys during early childhood, thereby stimulating the gradual transition towards top-down forms of self-regulation with increasing age (Bernier et al., 2010).

### 4.4 Strengths, limitations, and future directions

The current study has several important strengths. First, the current study had a relatively large sample, which enhances the statistical power of the analysis and the overall accuracy of the findings. Second, the use of two different measurements of self-regulation allowed for a more comprehensive understanding of the neural mechanisms underlying both top-down and bottom-up forms of self-regulation in young children. Third, little research has been done on the maturational changes in theta power. Therefore, the current study extends prior work by examining both the development of baseline frontal alpha and theta power in relation to variations in child self-regulation. Fourth, in addition to previous longitudinal research in the field, the current study accounted for age differences at each measurement wave and considered that frequency bands evolve with age. This provided a new perspective on the developmental trajectories of frontal alpha and theta power during infancy and early childhood.

However, the findings must be viewed in light of some limitations. First, children in the current study were predominantly from families from higher socioeconomic backgrounds, which limits the generalizability of the findings (Miller-Cotto et al., 2022). For example, as a result of this homogeneity in socioeconomic status, children demonstrated relatively high levels of self-regulation. In addition, given the low variability in the growth curve models of frontal alpha power, caution should be taken about making generalizations to more diverse populations. Future research should strive to include participants from a broader range of socioeconomic backgrounds and with varying levels of frontal EEG power and child self-regulation to enhance the generalizability of our insights.

Second, there is a lack of knowledge about the frequency boundaries of meaningful EEG bands, leading to a great discrepancy in opinions on the alpha and theta frequency ranges in young children. Although the age-adjusted frequency bands in the current study were applied in line with previous research (Marshall et al., 2002, Klimesch, 1999, Orekhova et al., 2006), the specific nature and function of the different EEG frequency bands remains unclear. Future studies could further examine the meaningful frequency boundaries of the alpha and theta rhythm, for instance by determining narrower frequency ranges (e.g., lower and upper bands) or defining the boundaries based on the individual peak frequencies (Klimesch et al., 2001, Marshall et al., 2002, Perone et al., 2018b). In addition, more research on the maturational changes in power ratio measures (e.g., theta/beta) and frontal asymmetry is needed, as this might offer a more comprehensive picture than examining frontal alpha and theta power alone (Perone et al., 2018b; Smith et al., 2016).

Third, a delay of gratification task was included in the current study to assess individual differences in child self-regulation. Since the ability to delay gratification typically emerges in children at around the age of three (Blair and Ursache, 2011, Garon et al., 2008), earlier measures of delay of gratification could not be integrated into the current research. For future studies, it may be beneficial to observe self-regulation using methods that are suitable for infants as well, such as eye-tracking to assess visual attention (Geeraerts et al., 2019) or A-not-B tasks (Bell, 2001, Morasch and Bell, 2011). These approaches allow for multiple measurement points in the assessment of self-regulation, offering insights into the foundational levels of self-regulation that might influence the levels of self-regulation during the preschool years. Moreover, incorporating multiple measurement points can facilitate the exploration of bidirectional relationships between frontal EEG activity and self-regulation during infancy and early childhood, that was outside the scope of the current study.

## 5 Conclusions

In sum, the current study sheds light on the neural mechanisms underlying child self-regulation by using age-adjusted EEG bands, providing a vital foundation for future developmental EEG research. Consistent with the study of Whedon et al. (2020), the findings imply that, while the neurobiological foundations of self-regulation are established during infancy, it is the maturation of the frontal alpha rhythm that contributes to variations in both top-down and bottom-up forms of self-regulation. A crucial finding was the higher scores on both measures of self-regulation in girls compared to boys, indicating that girls may reach the developmental milestones in both top-down and bottom-up mechanisms of self-regulation earlier than boys (Blair and Ursache, 2011, Nigg, 2017). Although sex differences in the maturational changes in frontal activity have not been revealed, these findings indicate that young boys might have a greater reliance on external regulatory mechanisms than girls (Bernier et al., 2010). More insight into the role of external regulators in brain maturation can help to implement programs and interventions aimed at establishing bottom-up self-regulatory skills early in life, in order to provide the necessary foundations for the emergence of top-down self-regulatory skills in the preschool period.

## CRediT authorship contribution statement

**Joyce Endendijk:** Writing – review & editing, Supervision, Methodology, Conceptualization. **Marissa Hofstee:** Writing – original draft, Software, Methodology, Formal analysis, Data curation, Conceptualization. **Maja Deković:** Writing – review & editing, Supervision, Methodology, Funding acquisition, Conceptualization. **Julie Vidal:** Writing – review & editing, Conceptualization. **Bauke van der Velde:** Writing – review & editing, Software, Data curation. **Jorg Huijding:** Writing – review & editing, Supervision, Methodology, Conceptualization.

## Data statement

The research was conducted in accordance with APA ethical standards in the treatment of the study sample. Ethical approval for the YOUth cohort study was provided by the Medical Research Ethics Committee of the University Medical Center Utrecht and informed consent was obtained from both parents at each wave. The materials, the syntax needed to reproduce the analyses, and the data that support the findings of the current study are available upon request: https://www.uu.nl/en/research/youth-cohort-study/data-access.

## Author Note

Declarations of interest: none. The research was conducted in accordance with APA ethical standards in the treatment of the study sample. Ethical approval for the YOUth cohort study was provided by the Medical Research Ethics Committee of the University Medical Center Utrecht and informed consent was obtained from both parents at each wave. This study was supported by a grant from the Dutch Ministry of Education, Culture, and Science, and the Netherlands Organization for Scientific Research (NWO): the Gravitation Program Consortium on Individual Development (NWO Grant no. 024.001.003).

## Declaration of Competing Interest

The authors declare that they have no known competing financial interests or personal relationships that could have appeared to influence the work reported in this paper.

## Footnotes

1 Due to the presence of a multilevel structure when using the TSCORES option in Mplus to model individually varying times of observation, standardized coefficients could not be obtained. As a result, unstandardized coefficients were reported.

2 In contrast to the lower AIC value, the unconstrained model showed a higher BIC value compared to the constrained model. In some cases, the BIC can be overly conservative and prefer simpler models over more complex models (Chen et al., 2017, Dziak et al., 2020). The Sample-Size Adjusted BIC (SABIC) is a modification of the traditional BIC. The SABIC tends to be less inclined to select under-fitted models across various sample sizes, thereby helping to prevent the rejection of more complex models that might be justifiable based on theoretical considerations (Chaurasia, 2022). In line with the AIC value, the SABIC value of the unconstrained model (*M* = − 2257.493) was lower compared to the SABIC value of the constrained model (*M* = − 2247.299). Therefore, the results of the unconstrained model were prioritized for further consideration.
