# Supplementary material for: Maturational changes in frontal EEG alpha and theta activity from infancy into early childhood and the relation with self-regulation in boys and girls
Source: Dev Cogn Neurosci. 2024 Sep 19;70:101445. doi: 10.1016/j.dcn.2024.101445 (PMC11460477; doi:10.1016/j.dcn.2024.101445)
Supplement: Supplementary file 1 — Supplementary material [file mmc1.docx]

**EEG analysis**
 The EEG recordings were analyzed in Matlab, using functions of the FieldTrip toolbox (Oostenveld et al., 2011). The original 2048 Hz data was down sampled to 512 Hz, using chip interpolation and band-pass filtered at 0.1-70 Hz with a two-way Butterworth filter. Cleaning of the EEG data was accomplished by removing trials that contained too high amplitudes (> 250 µV); contained jumps (detected with ft_jump_removal.m); were excessively non-normal (kurtosis > 7); contained flatlining electrodes (inverse of variance > 0.1); or contained excessive noise (variance > 1500). If more than two bad channels were found (i.e., more than 40% of the signal in the channel contained artifacts), the EEG recording of the child was considered as missing data.
 The cleaned data for each child was bandpass filtered into 9 frequency bands: delta (0.1-2.9 Hz), infant theta (3-5.9 Hz), toddler theta (4–6.9 Hz), adult theta (4 – 7.9 Hz), infant alpha (6–8.9 Hz), toddler alpha (7-9.9 Hz), adult alpha (9-11.9 Hz), beta (12–24.9 Hz), and gamma (25–45 Hz). In the current study, the focus was on the alpha and theta frequency rhythms, as these rhythms are thought to reflect cognitive performance in the developmental EEG literature (e.g., Whedon et al., 2020; Klimesch, 1999).
 Given that the boundaries of these frequency bands are known to shift from infancy to early childhood, including higher spectral peaks around 4 years of age (Marshall et al., 2002; Perone et al., 2018b), age-adjusted alpha and theta frequency ranges were applied to better capture the cortical rhythms at the different waves. More specifically, at T1 and T2, the EEG data were bandpass filtered into the infant frequency bands. At T3, the EEG data of children under 4 years of age were bandpass filtered into the toddler frequency bands, whereas the EEG data of children above 4 years of age were bandpass filtered into the adult frequency bands. The decision to use an adult alpha and theta frequency band for children aged 4 and above ensures consistency with the established literature and EEG standards used in older children and adults (Cavanagh & Frank, 2014). That is, previous studies that use adult bands in older children generally use 8-12 Hz and 4-8 Hz for alpha and theta respectively (Lo et al., 2013; Perone et al., 2018). All children who exhibited distinct alpha and theta peaks had their individual peak frequencies align with their age-adjusted frequency bands.
 Spectral analysis of the calibration signal and computation of power values was accomplished in line with the studies of Jones et al. (2015) and Van der Velde et al. (2021). That is, the cleaned EEG data were subjected to a Fast Fourier Transform (FFT) in Matlab. FFT produces the average power spectra for all electrodes over the time period that the signal was acquired by using range from filtering and frequency analysis to power spectrum estimation (Shaker, 2006). Subsequently, the power values were transformed using the natural log (ln) to normalize the distribution. EEG power can be obtained across all electrode sites on the scalp, but it is commonly aggregated into smaller clusters that focus on specific regions of interest (Perone et al., 2018). In line with the study of Whedon et al. (2020), a frontal EEG power composite was calculated by averaging the frontal (Fp1, Fp2, F3, F4, F7, F8) power values, to create a general EEG power value over the broader frontal scalp area. In addition, normalized power was calculated by dividing the power values in the frontal scalp area by total power for each child. Specifically, we divided the power of the frontal area by the total power across the entire scalp within both the alpha and theta frequency bands. This was done to assess the power in the frontal region relative to the overall brain activity. The normalized alpha and theta power composites were used for further analysis.

**Age ranges**
The YOUth cohort chose to use a flexible design, in which repeated measurements for all children with a fixed time interval were incorporated. The three time points were chosen based on the developmental perspective of self-regulation, with a rapid growth in co-regulated behaviors during the first year of life and the emergence of more advanced cognitive forms of self-regulation, such as executive functioning, around the preschool period (Garon et al., 2008; Hendry et al., 2016; Kopp, 1982). However, not all children were measured at the same age (3-years age ranges). The main benefit of this flexible design is that it provides more detailed information that covers the range of typical development over multiple time points (Onland-Moret et al., 2020). Specifically, this approach allows researchers to capture the variability in developmental trajectories and provides a more comprehensive understanding of how EEG and SR evolve throughout early childhood.
