# Supplementary material for: Maturational changes in frontal EEG alpha and theta activity from infancy into early childhood and the relation with self-regulation in boys and girls
Source: Dev Cogn Neurosci. 2024 Sep 19;70:101445. doi: 10.1016/j.dcn.2024.101445 (PMC11460477; doi:10.1016/j.dcn.2024.101445)
Supplement: Supplementary file 2 — Supplementary material [file mmc2.docx]

**Coding of gift-delay videos** Following Kochanska et al. (2000), a score ranging from 1 to 5 was assigned to the behaviors of the child, based on the degree of restraint the child exhibited. Higher scores indicated longer delaying ability (1 = child takes gift out of the bag; 2 = child puts hand in the bag; 3 = child opens the bag and peeks into it; 4 = child touches the bag, but did not look into it or child looks into the bag, but did not touch it; 5 = child does not touch the bag or looks into the bag). For instance, when a child initially only touched the bag but later took the gift out of the bag, a score of 1 was assigned to the behavior of the child. In addition, the latencies (in seconds) to the assigned behaviors of the children were coded, with latency scores ranging from 0 (immediately) to 180 (never). Scores of the behaviors of the child and the latencies in seconds were significantly correlated (*r* = .46). Therefore, in line with previous studies (e.g., Carlson & Moses, 2001; Merz et al., 2017; Kochanska et a., 1996), both scores were first standardized and then averaged to create a single score for the ability to delay gratification.

**Training of coders**
 Before the start of the current study, an expert in the coding system trained two coders with 3 weekly sessions of two hours each. After the initial training session, during which the coders practiced coding the videos, the coders independently coded 5 and 10 practice videos at home, respectively. In the other two training sessions, the codings of the videos were discussed in detail. Disagreements between the coders were resolved through discussion until consensus was reached. After the three training sessions, the inter-rater agreement of the coders was examined by using the intra-class correlation coefficient (ICC), based on the double coding of 25 videos.
