# Supplementary material for: Maturational changes in frontal EEG alpha and theta activity from infancy into early childhood and the relation with self-regulation in boys and girls
Source: Dev Cogn Neurosci. 2024 Sep 19;70:101445. doi: 10.1016/j.dcn.2024.101445 (PMC11460477; doi:10.1016/j.dcn.2024.101445)
Supplement: Supplementary file 3 — Supplementary material [file mmc3.docx]

**Sensitivity analyses**

Knowledge on the differences between age-adjusted and non-age-adjusted frequency ranges is limited, since previous studies on the link between maturational changes in EEG activity and cognitive development have solely used non-age-adjusted frequency ranges (MacNeill et al., 2018; Whedon et al., 2020) or individually adjusted frequency ranges in different developmental periods (Perone et al., 2018b). To fill in this gap, an exploratory aim of the current study was to examine the differences between age-adjusted frequency ranges and non-age-adjusted frequency ranges when determining the maturational changes in frontal alpha and theta activity.
 In order to explore the differences between age-adjusted frequency ranges and non-age-adjusted frequency ranges, sensitivity analyses were performed. That is, beside the latent growth curve models with the age-adjusted frequency ranges, the latent growth curve models for both the alpha and theta rhythm were reanalyzed with the infant frequency ranges at all three time points.

**Results**

**Descriptive statistics** The means and standard deviations for boys and girls are presented separately in Table S1. Boys and girls did not significantly differ in their frontal infant alpha activity at T3, *t*(328) = -1.908, *p* = .057, as well as in their frontal infant theta activity at T3, *t*(328) =-0.218, *p* = .414. **Direct relations between the study variables** Results of the Pearson correlations are presented in Table S2. The results showed that, similar to the results regarding the age-adjusted frequency ranges, higher levels of frontal infant alpha power at T3 were associated with higher levels of both the ability to delay gratification and effortful control at T3. However, whereas variations in the age-adjusted frequency range of frontal theta power at T3 were negatively related to the ability to delay gratification at T3, no relations were found between the non-age-adjusted (i.e., infant theta) frequency range at T3 and the ability to delay gratification at T3. Also, no relations were found between frontal infant theta power at T3 and effortful control at T3.  **Developmental changes in frontal EEG activity** To assess the changes in frontal EEG activity over time, unconditional latent growth curve models for frontal infant alpha power and frontal infant theta power were modelled. Regarding the infant alpha rhythm, the results of the unconditional models showed a small but significant increase in frontal power over time (slope = 0.006, *p* = <.001). Children did not significantly differ in their initial levels of frontal infant alpha power (intercept = 0.004, *p* = .171), as well as their rate of change over time (slope = 0.000, *p* = .794). With regard to the infant theta rhythm, children showed a small developmental increase in frontal power from infancy into the preschool period (slope = 0.015, *p* = <.001). In addition, children significantly differed in both the initial levels of frontal infant theta power (intercept = 0.007, *p* = <.001) and their rate of change over time (slope = 0.000, *p* = .004).
**Relations between frontal EEG activity and self-regulation** Subsequently, the relations of the intercept and linear slopes of frontal infant alpha and frontal infant theta power with both the ability to delay gratification and effortful control were examined in two separate conditional models. The corresponding results are reported in Table S3. After fixing the variance of the intercept and the slope of frontal infant alpha power to zero, the model results showed that increases in frontal infant alpha from infancy into the preschool period were associated with greater abilities to delay gratification, but not with effortful control. With regard to the infant theta rhythm, the developmental changes were unrelated to both the ability to delay gratification and effortful control.
**Differences between boys and girls** The investigation of the differences between boys and girls with the use of multi-group analyses encountered convergence issues. To facilitate a comparison of model outcomes between boys and girls, separate growth curve models were therefore run for boys and girls and the results of both models were compared. Findings from the unconditional growth models revealed that frontal alpha power and frontal theta power showed an increase over time in both boys and girls (see Table S4). Regarding the alpha rhythm, there were no significant differences in terms of the initial levels and growth rates over time in both boys and girls. Concerning the theta rhythm, girls showed significant differences in both the initial levels of frontal theta power and the growth rate over time. In contrast, boys only showed a significant difference in their initial levels of frontal theta power. Subsequently, the maturational changes in frontal alpha and theta power did not show any significant associations with either of the two self-regulation measures in both boys and girls (see Table S5).

**Table S1.***Descriptive Statistics for Boys and Girls*

|  | Boys (*N* = 210) | | Girls (*N* = 232) | |
| --- | --- | --- | --- | --- |
|  | *M* | *SD* | *M* | *SD* |
| 1. Frontal infant alpha power T1 | 0.88 | 0.16 | 0.91 | 0.17 |
| 2. Frontal infant alpha power T2 | 0.76 | 0.17 | 0.77 | 0.14 |
| 3. Frontal infant alpha power T3 | 0.89 | 0.15 | 0.89 | 0.13 |
| 4. Frontal infant theta power T1 | 0.75 | 0.12 | 0.78 | 0.12 |
| 5. Frontal infant theta power T2 | 0.85 | 0.11 | 0.86 | 0.10 |
| 6. Frontal infant theta power T3 | 1.03 | 0.14 | 1.00 | 0.11 |
| 7. Delay of gratification behavior | 3.60 | 1.29 | 3.91 | 1.16 |
| 8. Observed latency score | 88.53 | 66.54 | 101.33 | 67.29 |
| 8. Effortful control | 5.11 | 0.61 | 5.27 | 0.63 |

*Note.* In order to enhance the interpretability of the descriptive statistics, the mean scores for both the behaviors during the delay of gratification task and the latency to exhibit this behavior are reported, rather than the composite score. In addition, the descriptive statistics of the non-transformed power values are reported.

**Table S2.***Correlations for Study Variables*

|  | 1 | 2 | 3 | 4 | 5 | 6 | 7 | 8 |
| --- | --- | --- | --- | --- | --- | --- | --- | --- |
| 1. Frontal infant alpha power T1 | — |  |  |  |  |  |  |  |
| 2. Frontal infant alpha power T2 | .20^**^ | — |  |  |  |  |  |  |
| 3. Frontal infant alpha power T3 | .13 | .27^**^ | — |  |  |  |  |  |
| 4. Frontal infant theta power T1 | .63^**^ | .24^**^ | -.02 | — |  |  |  |  |
| 5. Frontal infant theta power T2 | .19^*^ | .55^**^ | .11 | .35^**^ | — |  |  |  |
| 6. Frontal infant theta power T3 | .03 | .14* | .64^**^ | -.01 | .24^**^ | — |  |  |
| 7. Delay of gratification (composite) | -.09 | -.06 | .17^**^ | -.03 | -.06 | .06 | — |  |
| 8. Effortful control | -.09 | -.03 | .13^*^ | -.15^*^ | -.08 | .07 | .14^**^ | — |

*Note.* ^*^*p* < .05. ^**^*p* < .01

**Table S3.**
*Unstandardized Estimates of the LGCM*

| Model parameters | *B* | *SE* | *p* | 95% CI |
| --- | --- | --- | --- | --- |
| Intercept frontal infant alpha power → delay of gratification | -5.84 | 6.29 | .353 | [-18.16, 6.49] |
| Slope frontal infant alpha power → delay of gratification | **1.83** | **0.92** | **.047** | **[0.02, 3.64]** |
| Intercept frontal infant alpha power → effortful control | -5.84 | 7.89 | .459 | [-21.30, 9.63] |
| Slope frontal infant alpha power → effortful control | 2.13 | 1.14 | .060 | [-0.09, 4.36] |
|  |  |  |  |  |
| Intercept frontal infant theta power → delay of gratification | -0.17 | 0.14 | .240 | [-0.45, 0.11] |
| Slope frontal infant theta power → delay of gratification | -2.44 | 3.05 | .423 | [-8.43, 3.54] |
| Intercept frontal infant theta power → effortful control | -0.20 | 0.19 | .310 | [-0.57, 0.18] |
| Slope frontal infant theta power → effortful control | 2.79 | 3.93 | .478 | [-4.91, 10.48] |

*Note.* Estimates presented in bold refer to statistically significant estimates.

**Table S4**

*Unstandardized Estimates of Intercepts and Slopes*

|  | Boys (*N* = 210) | | | Girls (*N* = 232) | | |
| --- | --- | --- | --- | --- | --- | --- |
|  | Estimate | *SE* | *p* | Estimate | *SE* | *p* |
| **Frontal alpha power** |  |  |  |  |  |  |
| Mean intercept | **-0.231** | **0.017** | **.000** | **-0.215** | **0.019** | **.000** |
| Variance intercept | 0.005 | 0.004 | .216 | 0.003 | 0.004 | .454 |
| Mean slope | **0.007** | **0.001** | **.000** | **0.006** | **0.001** | **.000** |
| Variance slope | 0.000 | 0.000 | .931 | 0.000 | 0.000 | .729 |
| **Frontal theta power** |  |  |  |  |  |  |
| Mean intercept | **-0.248** | **0.012** | **.000** | **-0.213** | **0.011** | **.000** |
| Variance intercept | **0.006** | **0.003** | **.017** | **0.007** | **0.002** | **.001** |
| Mean slope | **0.017** | **0.001** | **.000** | **0.013** | **0.001** | **.000** |
| Variance slope | 0.000 | 0.000 | .122 | **0.000** | **0.000** | **.006** |

*Note.* Estimates presented in bold refer to statistically significant estimates.

**Table S5.**
*Unstandardized Estimates of the LGCM of Boys and Girls Separately*

|  | Boys (*N* = 210) | | | Girls (*N* = 232) | | |
| --- | --- | --- | --- | --- | --- | --- |
|  | *B* | *SE* | *p* | *B* | *SE* | *p* |
| Intercept frontal alpha power → delay of gratification | -4.95 | 9.59 | .606 | -6.71 | 8.61 | .436 |
| Slope frontal alpha power → delay of gratification | 1.34 | 1.34 | .314 | 2.28 | 1.23 | .065 |
| Intercept frontal alpha power → effortful control | -6.96 | 11.09 | .530 | -0.58 | 12.08 | .962 |
| Slope frontal alpha power → effortful control | 2.36 | 1.45 | .104 | 1.67 | 1.72 | .333 |
|  |  |  |  |  |  |  |
| Intercept frontal theta power → delay of gratification | -0.17 | 0.22 | .433 | -0.12 | -0.20 | .538 |
| Slope frontal theta power → delay of gratification | -2.62 | 4.61 | .570 | -0.22 | 4.58 | .962 |
| Intercept frontal theta power → effortful control | -0.22 | 0.38 | .567 | -0.16 | 0.22 | .462 |
| Slope frontal theta power → effortful control | 6.78 | 7.08 | .338 | 1.30 | 5.37 | .809 |

*Note.* Estimates presented in bold refer to statistically significant estimates.

**Discussion**

In sum, both infant alpha and theta power showed a small but significant increase over time. In addition, the model results showed that increases in frontal infant alpha from infancy into the preschool period were associated with greater abilities to delay gratification, but not with effortful control. With regard to the infant theta rhythm, the developmental changes were unrelated to both the ability to delay gratification and effortful control. Furthermore, the maturational changes in frontal alpha and theta power did not show any significant associations with either of the two self-regulation measures in boys and girls separately.

**Non-age-adjusted frequency ranges**

Consistent with prior research using non-age-adjusted frequency ranges (Whedon et al., 2020), the use of the infant alpha band at all three waves resulted in an increase in frontal alpha power over time. This could potentially be attributed to the natural expansion of brain activity towards higher frequencies with increasing age (Cuevas & Bell, 2022; Marshall et al., 2002). Surprisingly, the use of the infant theta band at all three waves also resulted in an increase in frontal theta power over time. This result deviates from the limited studies reporting a decrease in theta activity during early childhood and highlights the complexity of examining maturational changes in brain structure and function in young children (Klimesch, 1999; Orekhova et al., 2006). One explanation might be the use of broad frequency ranges, in which the upper-theta rhythm will increasingly demonstrate properties similar to the alpha rhythm (Orekhova et al., 2006). Yet, the maturational changes in theta power during early childhood are largely unknown and further investigations into the complexities surrounding age-related changes in frontal theta activity are needed.

While the maturational changes in frontal alpha power demonstrated associations with both measures of self-regulation when age-adjusted frequency ranges were used, the maturational changes in frontal alpha power, using the infant alpha rhythm throughout all three waves, were only linked to the ability to delay gratification in the preschool period. In addition, there were no relations between maturational changes in frontal theta power and child self-regulation with the use of non-age-adjusted frequency ranges. When the conditional growth models for boys and girls were examined separately, boys and girls both showed an increase in baseline frontal alpha and theta power over time. However, these maturational changes in frontal alpha and theta power did not show any significant associations with either of the two self-regulation measures in both boys and girls. Therefore, in comparison to the use of age-adjusted frequency rhythms, fewer associations with self-regulation in the preschool period were observed with the use of an infant rhythm throughout all three waves.

The different findings between age-adjusted and non-age-adjusted frequency rhythms underline the importance of considering age-appropriate analytical approaches when studying the maturational changes in brain activity during early childhood. Based on the findings of the current study, the effect of the increase in peak frequencies on the overestimation of alpha activity or underestimation of theta activity appears to be reduced by the application of age-adjusted frequency bands (Marshall et al., 2002; Perone et al., 2018b). Moreover, given the link with variations in both measures of child self-regulation, age-adjusted frequency bands seem to better capture the maturational changes in the frontal cortex compared to non-age-adjusted frequency bands (for a review, see Klimesch, 1999; Orekhova et al., 2006). However, more research is needed to further examine the differences between age-adjusted and non-age-adjusted approaches, to advance our understanding of the developmental brain changes during infancy and early childhood.
